# Network analysis suggests changes in food web stability produced by bottom trawl fishery in Patagonia

**DOI:** 10.1038/s41598-022-14363-y

**Published:** 2022-06-27

**Authors:** Manuela Funes, Leonardo A. Saravia, Georgina Cordone, Oscar O. Iribarne, David E. Galván

**Affiliations:** 1 Instituto de Investigaciones Marinas y Costeras (IIMyC-CONICET), Rodriguez Peña 4046 Nivel 1, B7602GSD Mar del Plata, Buenos Aires Argentina; 2 Centro Austral de Investigaciones Científicas del Consejo Nacional de Investigaciones Científicas y Técnicas (CADIC-CONICET), Bernardo Houssay 200, V9410CAB Ushuaia, Tierra del Fuego Argentina; 3 Centro para el Estudio de Sistemas Marinos-Consejo Nacional de Investigaciones Científicas y Técnicas (CESIMAR-CONICET), Bv. Almirante Brown 2915, U9120ACV Puerto Madryn, Chubut Argentina; 4 Instituto de Ciencias, Universidad Nacional de General Sarmiento, J.M. Gutierrez 1159 (1613), Los Polvorines, Buenos Aires Argentina

**Keywords:** Ecology, Ecological networks

## Abstract

Demersal fisheries are one of the top anthropic stressors in marine environments. In the long term, some species are more vulnerable to fishery impacts than others, which can lead to permanent changes on the food web. The trophic relationships between predator and prey constitute the food web and it represents a network of the energy channels in an ecosystem. In turn, the network structure influences ecosystem diversity and stability. The first aim of this study was to describe for the first time the food web of the San Jorge Gulf (Patagonia Argentina) with high resolution, i.e. to the species level when information is available. The San Jorge Gulf was subject to intense fisheries thus our second aim is to analyse the food web structure with and without fishery to evaluate if the bottom-trawl industrial fishery altered the network structure and stability. We used several network metrics like: mean trophic level, omnivory, modularity and quasi-sign stability. We included these metrics because they are related to stability and can be evaluated using predator diets that can weight the links between predators and prey. The network presented 165 species organized in almost five trophic levels. The inclusion of a fishery node adds 69 new trophic links. All weighted and unweighted metrics showed differences between the two networks, reflecting a decrease in stability when fishery was included in the system. Thus, our results suggested a probable change of state of the system. The observed changes in species abundances since the fishery was established, could represent the state change predicted by network analysis. Our results suggests that changes in the stability of food webs can be used to evaluate the impacts of human activity on ecosystems.

## Introduction

Fisheries are an important human activity and they can be found in almost every coastal system worldwide^1^. They are an essential source of animal protein for human consumption^2^ and employ a quarter of a billion people^3^. On the other hand, demersal fisheries are one of the top anthropic stressors in marine environments^4^, capable of modifying the habitat and its associated biological community^5^. The selectivity of the fishing gear, together with organism traits such as body size, lifespan and habits determine how vulnerable species are to fishing activity. Fisheries, in the long-term, can alter the abundance and diversity of species^6^ which could change trophic relationships and the trophic level of predators^7–9^, modifying the structure of the ecosystem.

Fisheries can change the relative energy demands of a community^10^ and alterations in the fluxes configuration can lead to changes in the stability of the food web^11^. Food webs characterize the trophic interactions (i.e. consumer-resource relationships) among species in an ecosystem^12^. The first step in food web analysis is to reconstruct the links between species, which describe network topology. The topology or the network structure has two components: nodes (standing for the species or groups of species), and links connecting nodes that represent the ecological interactions between the species. To describe and compare food webs, a useful approach is through network metrics related to stability and resilience of the system, like the level of omnivory^13^, the mean trophic level^14^, modularity^15^ and other metrics^16^. However, an accumulating body of evidence suggests that the relationship between structural properties and stability can only be understood if the strength of interactions are considered^17–19^.

**Figure 1:**
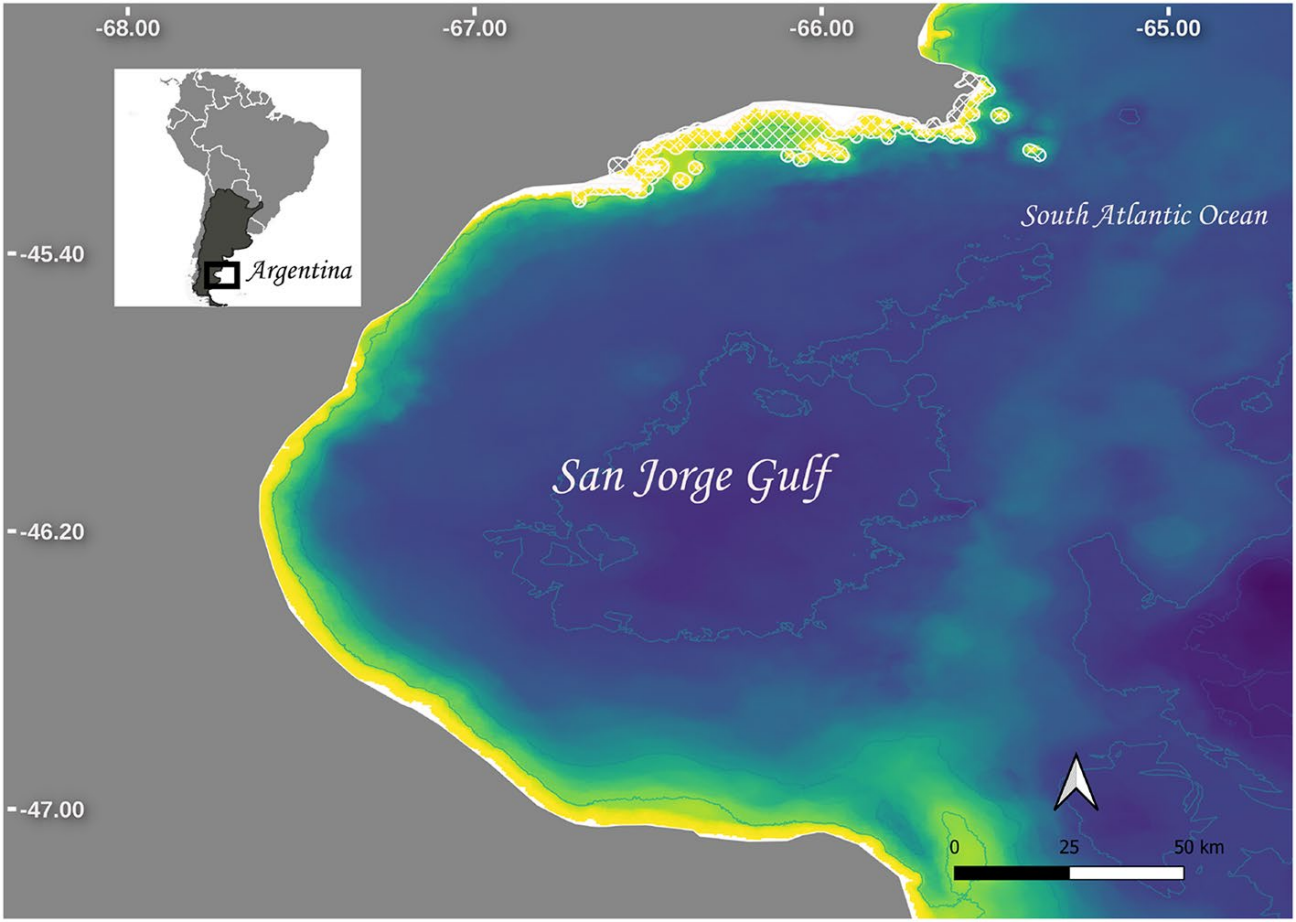
Study site location in Patagonia Argentina, South Atlantic Sea. Marine protected Area, “Patagonia Austral”, is marked in white. Map genereted with QGIS version 3.24.2 ‘Tisler’ from https://qgis.org/.

Bottom-trawl fisheries in northern Patagonia have mainly developed since the late 70s^20^. Main fishing targets are the Argentine hake; *Merluccius hubbsi*, and the Argentine red shrimp; *Pleoticus muelleri*, being the shrimp fishery the biggest crustacean fishery on the south occidental Atlantic in terms of abundance and revenues^20^. For the past 30 years, the main fishing activity in northern Patagonia has taken place in San Jorge Gulf (SJG), a semi-enclosed basin of approximately 230 km of latitudinal opening and approximately 150 km longitudinal wide (Fig. 1). SJG is a particularly productive area in Argentina’s waters where the fisheries coexist with big aggregations of marine mammals and seabirds’ colonies, oil extraction and touristic activity^21^. The local fish assemblage has low redundancy, where each ecological role was accomplished, on average, by one species^22^. Likewise, it was reported that bottom trawl fishery erodes fish functional diversity, leading towards homogenization^22^. In the shrimp fishery, 81 species are incidentally caught. However, the hake was described as “dominant” or “abundant” species in almost 60% of the catches^20^. As store space is limited and shrimp is better priced than hake, the latter was also dominant in discards composition^20^. Fishes, birds, marine mammals and crustaceans of the area were reported to feed on discards^23–26^. Discard practice makes available a surplus of food to scavengers but also to top-predators. Such surplus of novel and predictable food item is particularly important for non diving bird, as the kelp gull *Larus dominicanus* and the albatross *Thalassarche melanophrys* which are the most important consumers of discards among seabirds^23^. Moreover, discards of hake, a demersal fish not accessible for non diving birds, was identified to be an important factor triggering their population increase observed since 80s’^23,27^. Although some insights were reported at the population level for certain species, how discard biomass impacts the local community remains unaddressed, and this phenomenon requires an integrated ecosystem approach, like the one offered by food web theory^16^.

Information on the functionality of SJG is scarce and its understanding has economic and social importance for the region. Therefore, it was set as a priority in a national research initiative aiming to promote an ecosystem based management of the resources^28^. In addition to this local interest, the theoretical description of the food web structure and its modifications by anthropic stressors are a fundamental question worldwide. This study aims to achieve a high-resolved food web description of SJG and evaluate if the bottom-trawl industrial fishery alters network structure and stability.

## Methods

### Data

We made a systematic search of all available studies on species and diets of marine animals of the area to build the SJG food web. The nodes of the network are the species, but in cases where there was not enough taxonomic information available, these were grouped as trophospecies.

We included stomach content analysis and direct observations studies. When the diet was not reported for SJG, studies conducted at neighbour areas were used. In these cases, we included only prey items reported for SJG. Meiofauna (organisms < 1 mm) and parasites were not included. After consulting more than 300 papers, 137 of them had useful information to build the SJG food web, and the resulting list of species and interactions were revised by experts who work on SJG (Available at https://www.zotero.org/groups/4664638/networksanjorgegulf/library and as a BibTeX file in the repository https://github.com/EcoComplex/NetworkGolfoSanJorge).

The consumer’s diet composition was estimated by the percentage of the wet weight of the prey. When wet weight information was not available, the number of organisms or the frequency of occurrence was used to assign the relative contribution of each prey. When none of those metrics were available, the diet was estimated based on the relative abundance of prey items in catch records, assuming capture reflects abundance. For those species whose prey are not subject to capture and no other data were available, the diet was estimated by consulting local experts. For each predator, the sum of total diet preferences has to equal one.

From all reported trophic links, we created two food web scenarios: non-fishing and fishing. Natural trophic interactions were included in the non-fishing scenario; i.e. without considering fishing influence. Two new nodes, “Fishery” and “Discard”, were added in the fishing scenario. The “Fishery” node act as a consumer of species caught by the trawl net. These species were identified in shrimp fisheries records^29^. The dominant bycatch species discarded is hake^20^. In this sense, the “Discard” node represents mainly hake discards. The “Discard” node act as a resource to scavengers and opportunistic species. We added a link between “Discard” and scavengers or opportunistic species when these species presented hake in their diet and are incapable of capturing hake alive^30–32^. This incapability is ruled by their predator capacities or by habitat constraints. For example, surface predators are not capable of feeding upon a demersal fish^33^. Discard consumption by species that regularly predate on hakes, like the sea lion^34^ or the penguin^35^, were not included in the fishing scenario because it was not possible to discern the source, natural or facilitated by the fishery, of this prey item.

**Figure 2:**
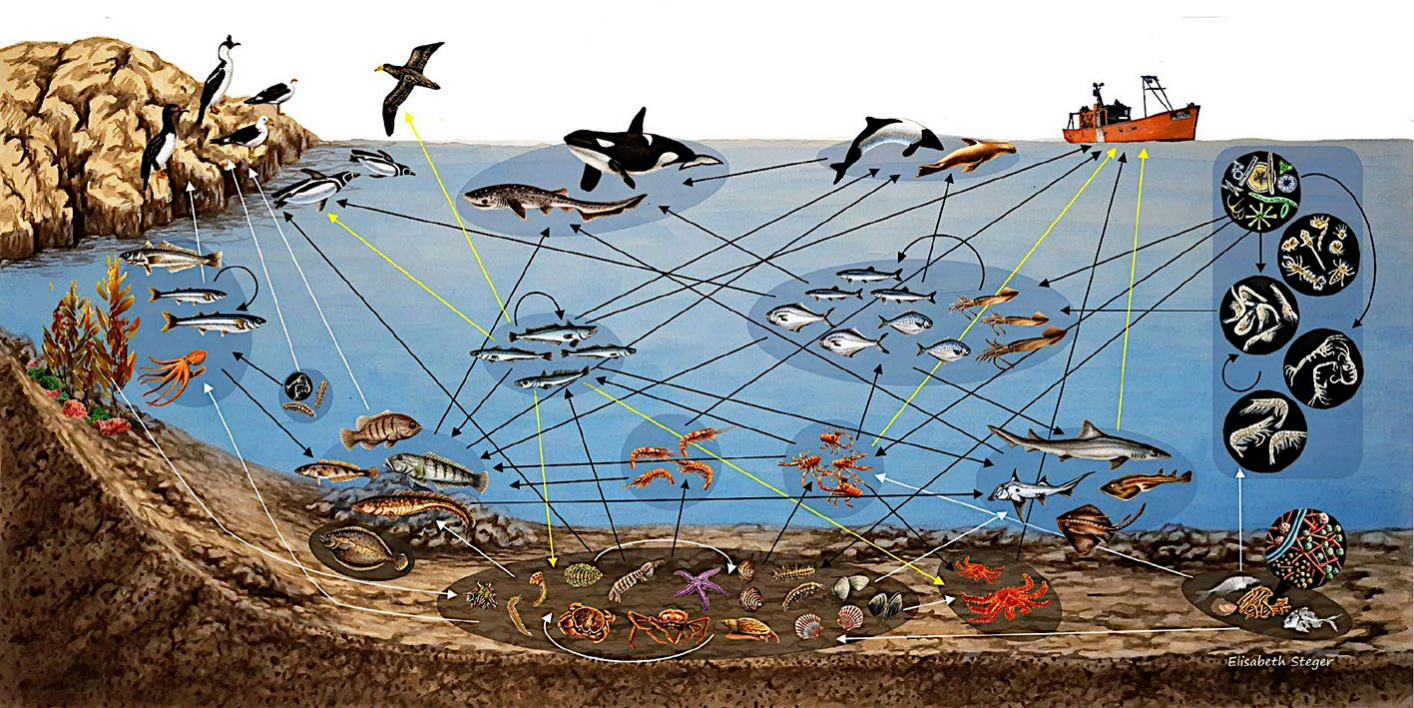
Simplified food web representation of the most important species in terms of the degree, relative biomass and taxon representation. Arrows represent energy and biomass fluxes direction, yellow arrows represent fluxes produced by the fishery activity (capture and discard consumption). The image was obtained with permission from^28^.

### Network metrics

To characterize the SJG food web, we used four network metrics in two versions: (1) unweighted: only considering the existence of a trophic relationship (links), in this case, all the links count equally. (2) Weighted: we used the consumption percentage of each prey to weigh the links. The latter gives more importance to the preferred prey. The metrics were: the mean trophic level (mTL); since it is among the most sensitive indicator of fishing pressure used in ecosystem-based fisheries management^36^ that can be calculated with our topological approach. Food webs with high mTL are supposed to be less stable^14^. On the contrary, lower mTL means that there are fewer steps between a species and a basal resource which indicates more energy-efficient system^37^. The level of Omnivory; defined as the percentage of nodes consuming at more than one trophic level^38^. Omnivory can have either a positive or negative influence on the stability of food webs, depending on the interaction strength. High levels of omnivory are always destabilizing, intermediate levels may stabilize food webs^39^. Omnivory is likely to persist at intermediate productivity levels and be more common in disturbed environments^13^. The modularity is the degree to which a subgroup of species interact more with themselves than with the rest of the nodes^15,40^. Higher levels of modularity presume higher stability because the compartmentalization prevents disturbances from spreading but it was proved beneficial only for perturbed ecosystems. We calculated the best compartment partition using a stochastic algorithm based on simulated annealing that allows maximizing modularity for directed and weighted networks without getting trapped in local maxima configurations^41^.

Finally, we calculated the quasi sign-stability (QSS) index, which is the proportion of stable networks using 10000 randomized Jacobians and keeping the predator prey structure fixed^42^. The formulas for the two versions of the metrics are explained in the Supplementary Methods.

### Analysis

Food web metrics were estimated for both food webs (with and without fishing activity) in order to assess their differences. Considering the fishery as a disturbance, we expected the food web in the non-fishing scenario to be shifted from an equilibrium state. And that shift would be reflected in the network metrics, comparing the fishing and non-fishing scenarios. For the comparison we performed 1000 randomizations (except for QSS, see above) using the curveball algorithm which maintains constant the number of prey and predators for each species, therefore keeping fixed the number of columns and rows in the adjacency matrix^43^. In the case of the weighted metrics additionally, the values of weights (diets) were randomized maintaining the column sum fixed on the weighted adjacency matrix (see formulas on the Supplementary Material). The distribution of the metrics were compared between the fished and non-fishing model using Anderson–Darling test^44^, and the effect size; obtained by dividing the median of the data by the pooled standard deviation. The effect size interpretation is arbitrary^45^ so we use it as a relative measure of the magnitude of differences between food webs’ metrics. Quasi sign-stability values were compared using a Chi-squared test.

All estimations were performed in R software, using the packages igraph, NetIndices, and Multiweb package^46^. The source code and data are available at https://github.com/EcoComplex/NetworkGolfoSanJorge and Zenodo 10.5281/zenodo.6627973.

**Table 1:** Degree values (i.e. number of total interactions of each node) for the top 23 species and trophoespecies of the system from the non-fishing scenario and from the fishing food webs.

Species or trophospecies	Fishing	Non-fishing
*Pleoticus muelleri*	58	56
*Munida gregaria*	57	55
*Illex argentinus*	48	47
*Amphipoda*	48	48
Fishery	47	–
*Polychaeta*	45	45
*Isopods*	38	38
*Engraulis anchoita*	37	36
*Octopus tehuelchus*	37	36
*Zearaja chilensis*	37	36
*Detritus*	35	35
*Pseudopercis semifasciata*	35	34
*Merluccius hubbsi*	34	34
*Mustelus schmitti*	32	31
*Enteroctopus megalocyathus*	30	30
*Bathyraja* spp.	29	27
*Patagonotothen* spp.	28	26
*Paralichthys* spp.	28	26
*Pterygosquilla armata armata*	27	26
*Raneya* spp.	27	26
*Phalacrocorax atriceps*	24	24
*Atlantoraja castelnaui*	23	23
DISCARD	23	–

## Results

The non-fishing food web contained 165 trophic nodes, from which 115 were species and 50 were trophospecies. Nodes were connected by 1015 trophic links (Supplementary Fig. S2). The percentage of top predators was 16%, intermediate species 78% and basal species 6%. The network presented almost five trophic levels (TL), considering the top predator *Orcinus orca* with the maximum TL of 4.9, closely followed by *Notorynchus cepedianus* and *Mirounga leonina* (Supplementary Fig. S2, Supplementary Table S1). The top three more connected nodes were the Argentine red shrimp *Pleoticus muelleri* , the squat lobster *Munida gregaria*, the Argentine squid and, with the same degree, Amphipoda grouped as trophospecies (Supplementary Fig. S2; Table 1). These nodes were all crustaceans, located in the middle of the trophic network in terms of trophic TL (3.0, 2.5, 3.6 and 2 respectively). See Fig. 2 for a simplified representation.

**Figure 3:**
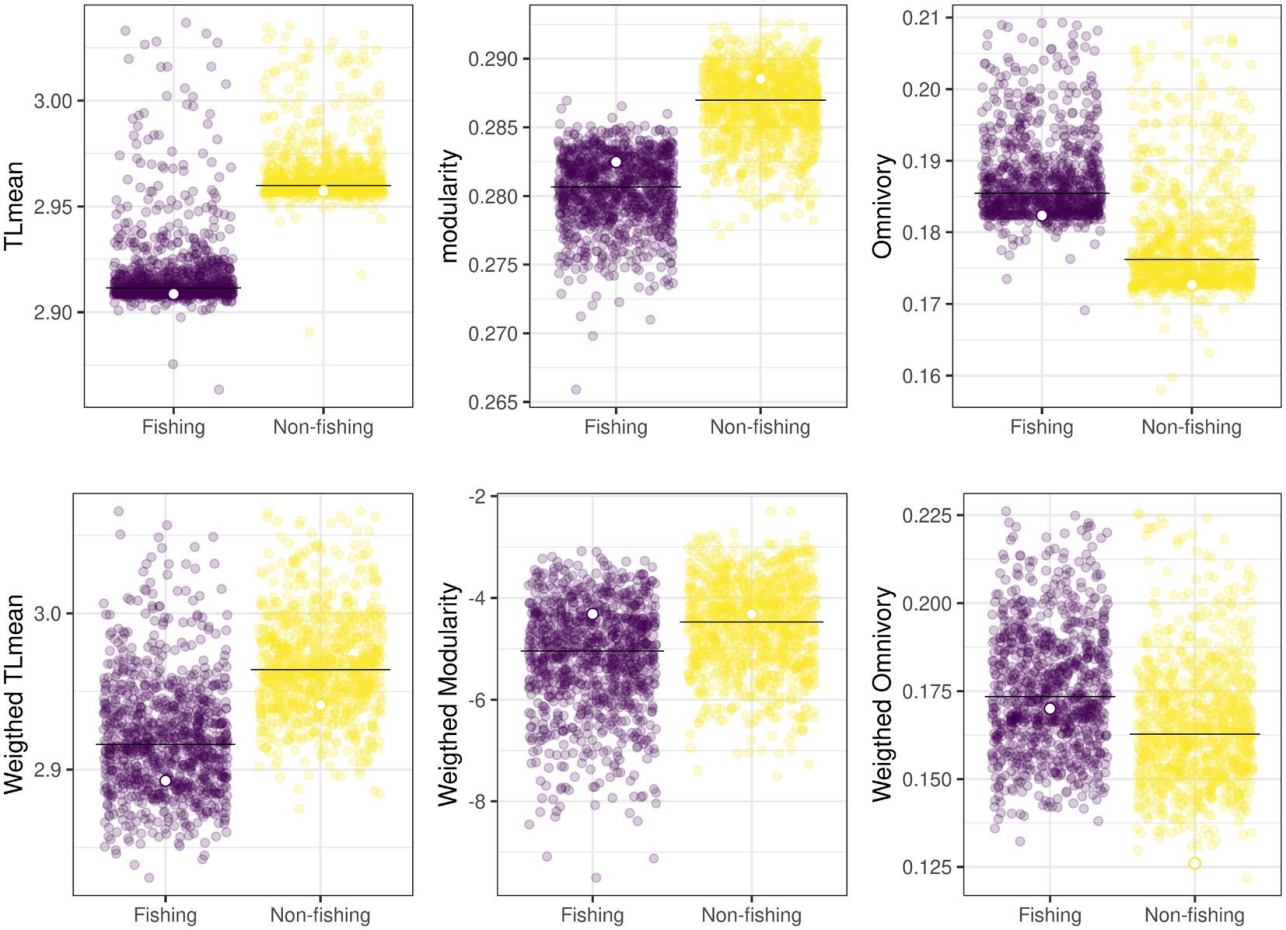
Comparison of the metrics for food-webs under fishing and non-fishing scenarios. The coloured points are randomizations of each food web keeping the number of links of each trophospecies constant, the weighted versions take into account the diets of the predators as link weight. The blank dots are the values of the empiric food webs, and the black lines the median of the randomized webs. Extreme values were eliminated from the plots for better visualization. These values were points below [Q1-(1.5)IQR] or above [Q3+(1.5)IQR], where Q1, Q3 are the first and third quantiles, and IQR is the interquartile range.

The fishing food web contained two extra trophic nodes and 69 extra trophic links, resulting in 167 nodes (115 species and 52 trophospecies), connected by 1084 links (Supplementary Fig. S1). The extra nodes were the fishery and the discards. The new links represented all new interactions enabled by fishing activity: the capture of species and the consumption of discards. The percentage of top predators was practically equal to the non-fishing food web (16%, intermediate species 78% and basal species 6%). The top predator was also *Orcinus orca* but its TL was a little lower: 4.8, *Notorynchus cepedianus* remained as the second predator but *Mirounga leonina* falls from 4.6 to 3.6 because it consumes discards (Supplementary Table S1). The degree of the species changes at most by 2 in the fishing food web, being the shrimp *Pleoticus muelleri* in the first place, the squat lobster *Munida gregaria* in the second and the squid *Illex argentinus* in third place (Table 1). These species were also located in the middle of the network in terms of TL: 3.0, 2.5, 3.6 respectively (Supplementary Table S1). The fishery had a TL= 4.3, ranked among the top ten predators in the system. The species caught by the fishery exhibited trophic levels between 2.0 (*Libidoclaea granaria*) and 4.5 (*Galeorhinus galeus*). On average, the fishery caught species with higher TL than the target species (red shrimp 3.0).

**Table 2:** Omnivory level, mean trophic level (mTL) and modularity values of the fishing and non-fishing model. For each metric, the median difference, pooled standard deviation and the magnitude of the effect size, estimated as the median difference over the pooled sd.

Type	Metric	Non-fishing median	Fishing median	Median difference	Pooled sd	Effect size
Unweighted	Omnivory	0.1768	0.1862	$-$ 0.0094	0.1017	$-$ 0.0921
	mTL	2.9606	2.9118	0.0488	0.0879	0.5546
	Modularity	0.2870	0.2807	0.0063	0.0041	1.5494
Weighted	Omnivory	0.1642	0.1754	$-$ 0.0113	0.7476	$-$ 0.0151
	mTL	2.9657	2.9172	0.0485	0.1942	0.2498
	Modularity	$-$ 4.4740	$-$ 5.0443	0.5703	1.0199	0.5591

**Figure 4:**
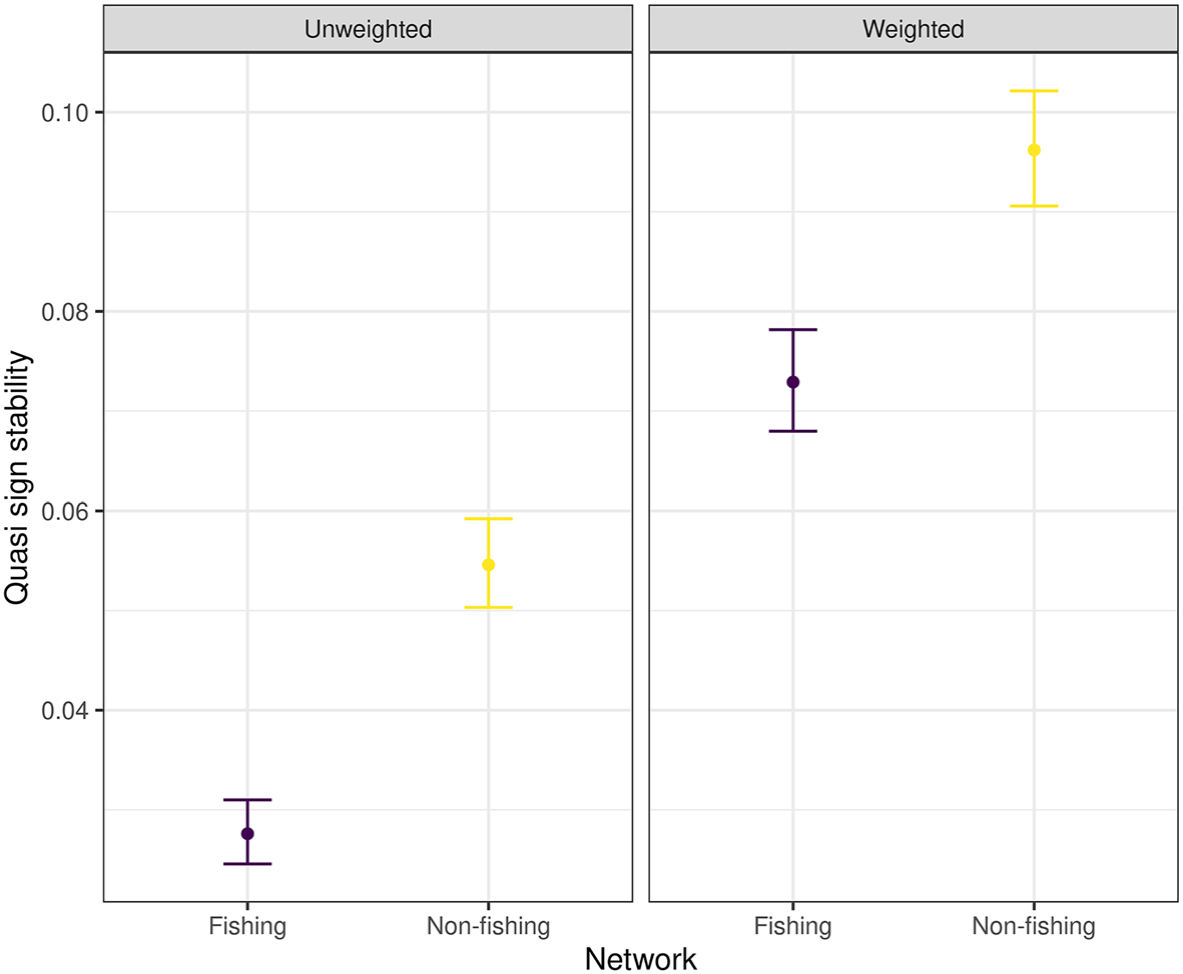
Quasi sign-stability (QSS) values of the fishing and non-fishing food webs. QSS is a measure of the capacity of the system to return to equilibrium after a perturbation, the lower it is the less capacity the system has. Unweighted values consider only the topological structure of the food web, the weighted values take into account the diets of the species. Values are presented with their standard deviation.

There are significant differences between the fishing and the non-fishing food webs topological metrics (mean TL, modularity and omnivory coefficient: Anderson Darling test *p =* 0.00). The biggest effect size in absolute value was observed for modularity and the smallest for omnivory (Table 2, Fig. 3). In terms of stability, the fishing food web was less stable than the non-fishing (Quasi sign-stability *p* < 2.2e−16, Fig. 4). The results for the diet weighted metrics were similar to the previous ones: all differences were significant and the effect sizes had the same ordering but were smaller than the unweighted ones. Regarding QSS, the differences between food webs were bigger for the unweighted case. Only for the weighted omnivory metric the empirical food web value falls outside the null model range (Fig. 3). This could be due to the randomization of predators’ diet. The weighted modularity was negative indicating that the link weights between the modules are larger than the weights inside the modules.

## Discussion

We found that fishery activities in the San Jorge Gulf (SJG) could reduce the stability of the food web. Recent results suggest that unweighted topological metrics can not detect either alterations in fluxes^47^ or changes in stability^19^. In our study, both the weighted and unweighted metrics showed the same pattern, with quasi sign-stability being the metric with the most straightforward interpretation that showed the lowering of stability.

The present study constitutes the first attempt to construct a high-resolved food web of the SJG ecosystem. We collected detailed information for several low, medium and high trophic level (TL) species, which resulted in a speciose food web between the most resolved ones^48^. SJG is a key feeding, reproductive and nursing area for the main lucrative fisheries in Argentina. The description of our network exposes the possible flows of matter and energy in the studied system, which is considered a fundamental requirement in the Ecosystem Approach of fisheries^49^. Also, our resulting networks are a key input to build an Ecosystem Services Framework, as described in Armoskaité et al.^50^.

The food web of SJG is a complex system with more than a hundred species and almost five TLs. It has several top predator populations which include many colonies of marine mammals, sea elephants, sea lions, dolphins, orcas, and also marine birds and sharks. The importance of top predators is given by their trophic function, and their loss affects the uniqueness of the system^51^. Diverse top predators populations are not frequently found in other coastal systems^52^ and their depletion is a common consequence of overexploitation^53–55^. In SJG, a Marine Protected Area “Patagonia Austral” was built to protect top predator colonies (Fig. 1)^21^ and some populations were reported to be increasing in numbers^56–58^. Other top predator populations like sharks have no formal protection and their populations were reported to be decreasing^59^.

Network analysis is a powerful tool to study ecosystems changes related to anthropogenic factors^60–62^. However, there are different interpretations of network metrics in the ecological context. One of the most famous controversies is the “complexity-stability” debate which still remains active^63–66^. In this sense, the role of omnivory in food web stability has also been a matter of debate. McCann and Hastings^67^ challenged the classical view of Pimm and Lawton^68^ by proposing that omnivory could be a stabilizer in food webs. Gellner and McCann^39^ showed that the role of omnivory in food web stability critically depends on interaction strength. Wootton^13^ reviewed various mechanisms whereby weak omnivorous links are frequently found in freshwater ecosystems. Although interactions strengths are key to food web dynamics, high values of omnivory are always destabilizing^39^. The level of omnivory was already high in the non-fishing system compared to other marine systems^48,69^ and increased in the fishing system. This is a consequence both of fishery consuming resources from different TL^70^ and of the discard consumption^71^. Thus, the increase in the omnivory level of the fishing network could lead to a decrease in ecosystem stability. Changes at modularity levels also have consequences on food web stability. The effect of modularity on food web stability is to buffer perturbations, with a stronger effect when the system is more complex and is subject to perturbations^72^. In this study, the food web in the fishing scenario presented higher levels of omnivory accompanied by lower levels of modularity. Both metrics point out towards a lower level of stability that is supported by the quasi sign-stability difference between networks.

Previous studies showed that a higher maximum TL is related to less stability, measured as Quasi sign-stability (QSS)^14^. We found that the non-fishing food web was more stable, measured by QSS with and without weights, with a higher mean trophic level (TL) and this result seems to contradict the previously mentioned work. More recent theoretical and empirical studies suggest that increasing the mean TL could increase stability^73,74^, and that there could be different responses depending on how the food web is regulated^75^. Besides that, the differences observed in our results are smaller than the ones studied in the work of Borrelli and Ginzburg, thus the TL does not seem to be related to stability unless exceedingly high values were reached^14^.

Regarding TL, results also showed that the mean TL of the food web was lower in the fishing scenario. Discard consumption was introduced as a basal node which methodologically forced a decrease in mean TL. On the other hand, the effect of the node “fishery” on the mean TL of the food web could not be predicted a priori. The described shrimp fishery is a particular case of a fishery which targets a species of TL = 3 and discards species of higher TL (discard composition was described to be dominated by hake)^20,33,35^. The node “fishery” had a TL of 4.4, being among the top ten predators. In accordance with our characterization of the fishery and its effects, a functional diversity description of the San Jorge gulf fish assemblages showed that demersal and benthic species bigger than 30 cm of total length with intermediate to high TLs were the most vulnerable to shrimp fishery^22^. Moreover, the sites with higher industrial trawling activity have assemblages with slightly lower mean TL^22^, coinciding with the overall effect of the fishery of the decrease in the mean TL.

The SJG food web was previously described using mass-balance trophic models (Ecopath with Ecosim)^76^. The length of the food web (i.e. maximum TL) resulted similar (4.5 with Ecopath and 4.9 with present approach). Also TL values of the most abundant nodes were in the same range of values compared with results of Ecopath^76^ and with other studies using stable isotope analysis^77^. Given estimations using stable isotope techniques are sensitive to trophic discrimination factors and tissue turnover rates^78–81^, value comparison is not straightforward. Present TL values were: anchovy *Engraulis anchoita* (3.1), shrimp *Pleoticus muelleri* (3.0) and squat lobster *Munida gregaria* (2.5). And according to Gaitán^77^ and Sanchez^76^ were 2.9–3.2, 3.4–2.6 and 3.1–2.2. Also, TL values similarities were found compared with particular studies of the area as the squat lobster: 2.5 in Funes^82^, and *Acanthistius patachonicus*: 3.7–3.8 this work and 3.9 in Funes^7^.

The inclusion of the fishery node and the availability of discard should produce a re-configuration of energy fluxes. Many changes have been reported from the beginning of fishery exploitation, 40 years ago. For example, a decrease in the size of landed hake of the southern stock between 1990 and 2013^83^, a decrease in the abundance of *Acanthistius patachonicus*, *Genypterus blacodes*, *Zearaja chilensis*, *Psamobatis spp* and *Sympterygia spp* in the catch of scientific surveys between 2005 and 2014^84^, and the recent increase in abundance of *Munida gregaria*^85,86^ that is also a connected species (a common prey). Thus, all these changes reflect an important modification of energy fluxes likely produced by the fishery. Specifically in an ecosystem as the SJG with low functional diversity, where each ecological role is covered by one or a few species, and the potential loss of a species compromises its ecological function^22^. We were unable to quantify these changes but most network metrics imply a decrease in stability that in turn suggest the system could change its state, and influence the ecosystem functioning.

In terms of degree, the network was primarily dominated by medium TL crustaceans; the shrimp, the squat lobster, and amphipods. The three trophic species are also dominant in abundance^87–89^, and are common prey to almost all fishes on the network^89–91^. The current importance of the squat lobster in the system^87^ does not match the description of the system performed 30 years ago^90^, and this change was reported for the entire area^85,92^. The squat lobster occurs in pelagic and benthic ecotypes, and in both environments it is important in terms of biomass^85,86^. Each ecotype feeds in the environment where it inhabits; pelagic forms feed on pelagic primary producers and benthic forms in benthic animals^82^. Besides we can only characterize the squat lobster as a high degree species, given the previously described results we could hypothesize that it captures energy and biomass from both environments and being a key prey item, could concentrate the energy flux between the base and the top of the system, connecting primary producers directly to top predators.

Overall results show that fisheries can decrease the stability of the food web. If high levels of omnivory are related to lower values of stability, omnivory could be a key feature to consider when using present information to inform management policies, since discard consumption, from low TL scavengers up to top predators, contributes greatly to the level of omnivory. Then, discard management strategies, like the Landing Obligation policy (e.g. Council Regulation No 1380/2013), could reduce stability differences between food web scenarios. Discard consumption by marine birds, like the kelp gull *Larus dominicanus* was described to subsidize its population increase^27^; after this population growth, problematic interactions with other species (like attacks to southern right whale *Eubalaena australis* calves) increased dramatically^93,94^. This last case demonstrates that undesirable indirect interactions can occur and due to the high number of interactions throughout the food web, single-species management strategies could produce unexpected results.

It is important to note that our results are based only on trophic interactions between species. But, species also interact with each other in non-trophic ways. Non-trophic interactions, such as mutualism, commensalistic, amensalistic, and competition have important consequences on ecosystem dynamics and stability^95–97^. For example, Mougi and Kondoh^98^ showed that antagonistic and mutualistic interactions can stabilize population dynamics. Also, Kéfi et al.^99^ showed that non-trophic interactions allow higher species persistence, higher total biomass, and enhance robustness to species loss in ecosystems. For these reasons, adding non-trophic interactions to the SJG food web may change our conclusions about the possible effects of the fishery on stability. The fishery probably disturbs non-trophic interactions between species, in consequence the impacts on the SJG ecosystem could be greater than suggested here. The next step to conquer is to incorporate non-trophic interactions into SJG network description and evaluate how the fishery may be altering them.

In spite of the limitations mentioned above, it is possible that our results are underestimating the effects of fishery exploitation in the ecosystem. As we only accounted for the consumption of hake by trophic guilds that cannot directly prey on it, we are aware that discard consumption affects more trophic guilds. In addition, other species besides hake are discarded, leading to more sources of underestimation of discarded consumption^26^. Regardless, our results showed how human activity can alter the structure and the stability of an ecosystem. This study took place in a relatively recently exploited area, compared to the Mediterranean, the North Atlantic or several fisheries of the Pacific, and therefore it maintains many “original” interactions worth to be described, understood and acknowledged before the system undergoes further changes.

## Supplementary Information

Supplementary Information.

## Data Availability

The source code and data is available at zenodo 10.5281/zenodo.6627973 and Github https://github.com/EcoComplex/NetworkGolfoSanJorge.
