# Supplementary material for: Network analysis suggests changes in food web stability produced by bottom trawl fishery in Patagonia
Source: Sci Rep. 2022 Jun 27;12:10876. doi: 10.1038/s41598-022-14363-y (PMC9237026; doi:10.1038/s41598-022-14363-y)
Supplement: Supplementary file 1 — Supplementary Information. [file 41598_2022_14363_MOESM1_ESM.pdf]

# Supplementary Information for the paper: Fisheries do not alter the structure but can change the stability of food webs

Funes Manuela Saravia Leonardo A. Cordone Georgina Iribarne Oscar O.
Galván David E.

## Formulas for the network properties

The food web can be represented by an adjacency matrix $A = (a_{ij})$ where $a_{ij} = 1$ if species $j$ predates on species $i$, else is 0, representing a link. This adjacency matrix could represent flows from $i \to j$ or weigths if $a_{ij}$ could be any real number. In this work we use both representations and the diets of predators as weigths. Then $k_i^{in} = \sum_j a_{ji}$ is the number of preys of species $i$ or its in-degree, alternatively it represents the in-flow (total flow from preys); as in our case these are diets we normalized it to sum 1. The quantity $k_i^{out} = \sum_j a_{ij}$ is the number of predators of $i$, its out-degree, or the out-flow (total flow to predators). The total number of edges is $L = \sum_{ij} a_{ij}$.

### Trophic Level

Based on[1] we first estimated the trophic level of a node $i$, defined as the average trophic level of its preys plus 1. That is:

$$tp_i = 1 + \frac{1}{k_i^{in}} \sum_j a_{ij} tp_j \tag{1}$$

where basal species that do not have preys (then $k_i^{in} = 0$) are assigned a $tp = 1$.

The mean trophic level is the mean of $tp_i$ over all species.

### Omnivory

Omnivory index for one species $i$ is defined based on the trophic level:

$$om_i = \frac{1}{k_i^{in}} \sum_j \left(tp_j - (tp_j - 1)\right)^2 a_{ij} \tag{2}$$

The index we use in this work is the mean of $om_i$ over all predators.

### Modularity

The index of modularity was defined as:

$$M = \frac{1}{2L} \sum_i \sum_j \left( a_{ij} - \frac{k_i k_j}{2L} \right) \delta_{ij} \tag{3}$$

where $\delta_{ij}$ is 1 if and only if the species $i$ and $j$ are on the same module and zero otherwise. $L$ is the total number of links and $k_i = k_i^{in} + k_i^{out}$, the total degree. For the weighted case the modularity has the following form:

$$M = \frac{1}{w}\sum_i \sum_j \left(a_{ij} - \frac{k_i^{in}k_j^{in}}{w}\right)\delta_{ij} \tag{4}$$

where $w = \sum_i k_i^{in}$ , which is the total weight.

### Quasi Sign-Stability

The Jacobian $J$, so-called community matrix,[2] represents the population-level effect of a change in one species' density on any other species, including the dependence on its own density (self-regulation), at an equilibrium. A system is locally stable if the Jacobian $J$ has all its eigenvalues negative, thus the maximal eigenvalue has to be less than zero for a system to be locally stable. The signs of the elements of $J$ are given by the predator-prey structure of the food web, but the magnitude of the elements are unknown. Following previous analysis,[3,4] we estimated the unknown magnitudes by drawing the predator-prey interactions from a uniform distribution ranging from -10 to 0, the prey-predator interactions from 0 to 0.1, and from 0 to -1 for the self-regulation effect. This implies that the predator effect on the prey is bigger than the effect of the prey on the predator, and that the self-regulation or self-damping effect, that scales the dynamic's return time, is generally smaller than the predator-prey effect. Other parametrizations are possible but they give very similar results (not shown). When the weights of the food web are present they are used as a factor for each of the magnitudes, so the distributions for predator-prey interacions goes from $-10 \times a_{ij}$ to 0, and the same is applied to the other types of interactions. The Quasi Sign-Stability (QSS) is the proportion of stable matrices, with negative maximal eingenvale, obtained for 10000 randomizations.

## References


1. Kones, J. K., Soetaert, K., van Oevelen, D. & Owino, J. Are network indices robust indicators of food web functioning? A Monte Carlo approach. *Ecological Modelling* **220**, 370–382 (2009).
2. May, R. M. *Stability and complexity in model ecosystems.* vol. 6 (Princeton university press, 1974).
3. Monteiro, A. B. & Faria, L. D. B. The interplay between population stability and food-web topology predicts the occurrence of motifs in complex food-webs. *Journal of Theoretical Biology* **409**, 165–171 (2016).
4. Borrelli, J. J. Selection against instability: Stable subgraphs are most frequent in empirical food webs. *Oikos* **124**, 1583–1588 (2015).


## Supplementary Table S1

Table S1: Trophic level calculated as specified in Supplementary methods for the two food webs.

| Species or trophospecies | Fishing | non-fishing |
|---|---|---|
| *Orcinus orca* | 4.8 | 4.936 |
| *Notorynchus cepedianus* | 4.481 | 4.591 |
| *Galeorhinus galeus* | 4.401 | 4.452 |
| *Otaria flavescens* | 4.376 | 4.421 |
| *Cephalorhynchus commersonii* | 4.357 | 4.38 |
| *Arctocephalus australis* | 4.312 | 4.406 |
| *Thalasseus maximus* | 4.289 | 4.3 |
| *Lagenorhynchus obscurus* | 4.279 | 4.297 |
| *FISHERY* | 4.268 | NA |
| *Squatina guggenheim* | 4.177 | 4.201 |
| *Globicephala melas edwardii* | 4.152 | 4.205 |
| *Sterna hirundinacea* | 4.083 | 4.095 |
| *Lagenorhynchus australis* | 4.068 | 4.089 |
| *Atlantoraja castelnaui* | 4.004 | 4.031 |

| Species or trophospecies | Fishing | non-fishing |
|---|---|---|
| *Phalacrocorax magellanicus* | 3.966 | 4 |
| *Dipturus chilensis* | 3.956 | 4.147 |
| *Seriolella punctata* | 3.948 | 4.116 |
| *Phalacrocorax atriceps* | 3.938 | 3.972 |
| *Spheniscus magellanicus* | 3.936 | 3.969 |
| *Parona signata* | 3.923 | 3.934 |
| *Phalacrocorax olivaceus* | 3.922 | 3.964 |
| *Percophis brasiliensis* | 3.864 | 3.866 |
| *Xystreuris rasile* | 3.823 | 3.967 |
| *Bassanago albescens* | 3.8 | 3.835 |
| *Zearaja chilensis* | 3.792 | 3.892 |
| *Squalus acanthias* | 3.791 | 3.809 |
| *Polyprion americanus* | 3.786 | 3.963 |
| *Macruronus magellanicus* | 3.761 | 3.785 |
| *Genypterus brasiliensis* | 3.76 | 4.273 |
| *Macronectes giganteus* | 3.758 | 3.948 |
| *Pseudopercis semifasciata* | 3.754 | 3.773 |
| *Thalassarche melanophris* | 3.752 | 4.008 |
| *Genypterus blacodes* | 3.746 | 3.85 |
| *Doryteuthis gahi* | 3.741 | 3.75 |
| *Paralichthys spp* | 3.71 | 3.863 |
| *Acanthistius patachonicus* | 3.697 | 3.798 |
| *Atlantoraja cyclophora* | 3.685 | 3.691 |
| *Psammobatis spp* | 3.661 | 3.734 |
| *Bathyraja spp* | 3.654 | 3.764 |
| *Stromateus brasiliensis* | 3.653 | 3.669 |
| *Mirounga leonina* | 3.623 | 4.571 |
| *Sebastes oculatus* | 3.618 | 3.726 |
| *Seriolella porosa* | 3.597 | 3.597 |
| *Mustelus schmitti* | 3.596 | 3.638 |
| *Cynoscion guatucupa* | 3.557 | 3.572 |
| *Illex argentinus* | 3.55 | 3.572 |
| *Scomber colias* | 3.545 | 3.547 |
| *Merluccius hubbsi* | 3.539 | 3.599 |
| *Trachurus lathami* | 3.5 | 3.5 |
| *Sprattus fuegensis* | 3.5 | 3.5 |
| *Larus atlanticus* | 3.5 | 3.506 |
| *Schroederichthys bivius* | 3.496 | 3.699 |
| *Eubalaena australis* | 3.482 | 3.5 |
| *Procellaria aequinoctialis* | 3.481 | 3.769 |
| *Eledone massyae* | 3.478 | 3.492 |
| *Raja flavirostris* | 3.473 | 3.699 |
| *Larus dominicanus* | 3.446 | 3.549 |
| *Callorhinchus callorynchus* | 3.412 | 3.648 |
| *Pinguipes brasilianus* | 3.372 | 3.372 |
| *Salilota australis* | 3.367 | 3.466 |
| *Sympterygia spp* | 3.333 | 3.623 |
| *Discopyge tschudii* | 3.321 | 3.451 |
| *Patagonotothen spp* | 3.32 | 3.542 |
| *Nemadactylus bergi* | 3.32 | 3.32 |
| *Enteroctopus megalocyathus* | 3.301 | 3.315 |
| *Myliobatis goodei* | 3.255 | 3.316 |
| *Triathalassothia argentina* | 3.25 | 3.25 |
| *Austrolycus laticinctus* | 3.25 | 3.25 |
| *Robsonella fontaniana* | 3.25 | 3.25 |
| *Raneya spp* | 3.25 | 3.25 |
| *Cheilodactylus bergi* | 3.229 | 3.229 |

| Species or trophospecies | Fishing | non-fishing |
|---|---|---|
| *Dules auriga* | 3.193 | 3.2 |
| *Tachyeres leucocephalus* | 3.184 | 3.184 |
| *Lithodes santolla* | 3.168 | 3.351 |
| *Iluocoetes sp* | 3.167 | 3.167 |
| *Semirossia tenera* | 3.167 | 3.167 |
| *Eleginops maclovinus* | 3.152 | 3.228 |
| *Pleurobranchaea maculata* | 3.144 | 3.144 |
| *Pterygosquilla armata armata* | 3.141 | 3.754 |
| *Urophycis brasiliensis* | 3.114 | 3.114 |
| *Octopus tehuelchus* | 3.111 | 3.111 |
| *Engraulis anchoita* | 3.1 | 3.1 |
| *Helcogrammoides cunninghami* | 3.1 | 3.1 |
| *Bovichthys argentinus* | 3.1 | 3.1 |
| *Cottoperca gobio* | 3.1 | 3.1 |
| *Mullus argentinae* | 3.063 | 3.062 |
| *Aphrodite* | 3 | 3 |
| *Congiopodus peruvianus* | 3 | 3 |
| *Pagurus comptus* | 3 | 3 |
| *Cnidaria* | 3 | 3 |
| *Themisto gaudichaudii* | 3 | 3 |
| *Gymnoscopelus nicholsi* | 3 | 3 |
| *Mnemiopsis leidyi* | 3 | 3 |
| *Agonopsis chiloensis* | 3 | 3 |
| *Asteroidea* | 3 | 3 |
| *Chaetognatha* | 3 | 3 |
| *Ramnogaster arcuata* | 3 | 3 |
| *Ribeiroclinus eigenmanni* | 3 | 3 |
| *Pleoticus muelleri* | 2.96 | 3.008 |
| *Carcinus maenas* | 2.76 | 2.792 |
| *Odontesthes spp* | 2.693 | 2.7 |
| *Odontocymbiola magellanica* | 2.667 | 2.667 |
| *Glyceridae* | 2.667 | 2.667 |
| *Doryteuthis sanpaulensis* | 2.583 | 2.583 |
| *Cyrtograpsus spp* | 2.571 | 2.571 |
| *Arbacia dufresnii* | 2.5 | 2.5 |
| *Heterosquilla polydactyla* | 2.5 | 2.5 |
| *Euphausiacea* | 2.5 | 2.5 |
| *Betaeus truncatus* | 2.5 | 2.5 |
| *Leucippa pentagona* | 2.5 | 2.5 |
| *isopods* | 2.5 | 2.5 |
| *Munida gregaria* | 2.464 | 2.501 |
| *Buccinanops globulosus* | 2.333 | 2.5 |
| *Artemesia longinaris* | 2.333 | 2.333 |
| *Ascidiacea* | 2.333 | 2.333 |
| *Austropandalus grayi* | 2.333 | 2.333 |
| *Echiurids* | 2 | 2 |
| *echinoids* | 2 | 2 |
| *Fissurella spp* | 2 | 2 |
| *chiton* | 2 | 2 |
| *Tegula patagonica* | 2 | 2 |
| *zooplankton* | 2 | 2 |
| *Ostracoda* | 2 | 2 |
| *Pseudoechinus magellanicus* | 2 | 2 |
| *Mysidacea* | 2 | 2 |
| *Renilla sp* | 2 | 2 |
| *Trophon geversianus* | 2 | 2 |
| *Bivalve* | 2 | 2 |

| Species or trophospecies | Fishing | non-fishing |
|---|---|---|
| *Perumytilus purpuratus* | 2 | 2 |
| *Balanus spp* | 2 | 2 |
| *Bryozoa* | 2 | 2 |
| *Hydrozoa* | 2 | 2 |
| *Libidoclaea granaria* | 2 | 2 |
| *Nereididae* | 2 | 2 |
| *Copepoda* | 2 | 2 |
| *Myxine spp* | 2 | 2 |
| *Nematoda* | 2 | 2 |
| *Holothurians* | 2 | 2 |
| *Peltarion spinosulum* | 2 | 2 |
| *Aequipecten tehuelchus* | 2 | 2 |
| *Porifera* | 2 | 2 |
| *Aulacomya atra* | 2 | 2 |
| *Ovalipes trimaculatus* | 2 | 2 |
| *Eurypodius latreillii* | 2 | 2 |
| *Nacella magellanica* | 2 | 2 |
| *ophiuroids* | 2 | 2 |
| *Leurocyclus tuberculosus* | 2 | 2 |
| *Mytilus edulis* | 2 | 2 |
| *Libinia spinosa* | 2 | 2 |
| *Majidae* | 2 | 2 |
| *Gasteropods* | 2 | 2 |
| *Peisos petrunkevitchi* | 2 | 2 |
| *Rochinia gracilipes* | 2 | 2 |
| *Polychaeta* | 2 | 2 |
| *Amphipoda* | 2 | 2 |
| *Decapoda* | 2 | 2 |
| *Eunicidae* | 2 | 2 |
| *detritus* | 1 | 1 |
| *POM* | 1 | 1 |
| *biofilm* | 1 | 1 |
| *DISCARD* | 1 | NA |
| *Diatomeas* | 1 | 1 |
| *coralline red algae* | 1 | 1 |
| *Cyanobacteria* | 1 | 1 |
| *Foraminifera* | 1 | 1 |
| *Macroalgae* | 1 | 1 |
| *phytoplankton* | 1 | 1 |

## Supplementary Table S2

Table S2: Species numbers reference for the food web figure S1 and S2

| Species | Fishing | Non-fishing |
|---|---|---|
| Aequipecten tehuelchus | 1 | 4 |
| Aphrodite | 2 | 5 |
| Arbacia dufresnii | 3 | 6 |
| Merluccius hubbsi | 4 | 22 |
| echinoids | 5 | 7 |
| Echiurids | 6 | 8 |
| Engraulis anchoita | 7 | 9 |
| Enteroctopus megalocyathus | 8 | 10 |
| Eunicidae | 9 | 11 |
| detritus | 10 | 2 |
| phytoplankton | 11 | 14 |
| zooplankton | 12 | 15 |
| POM | 13 | 16 |
| Nereididae | 14 | 17 |
| Polychaeta | 15 | 3 |
| biofilm | 16 | 18 |
| Mytilus edulis | 17 | 19 |
| Doryteuthis gahi | 18 | 20 |
| Illex argentinus | 19 | 21 |
| Munida gregaria | 20 | 23 |
| Cynoscion guatucupa | 21 | 24 |
| Patagonotothen spp | 22 | 25 |
| Trachurus lathami | 23 | 26 |
| Pleoticus muelleri | 24 | 27 |
| Pterygosquilla armata armata | 25 | 28 |
| Raneya spp | 26 | 29 |
| Sebastes oculatus | 27 | 30 |
| Bivalve | 28 | 31 |
| Artemesia longinaris | 29 | 32 |
| Atlantoraja castelnaui | 30 | 33 |
| Dules auriga | 31 | 34 |
| Libidoclaea granaria | 32 | 35 |
| Libinia spinosa | 33 | 36 |
| Mullus argentinae | 34 | 37 |
| Mustelus schmitti | 35 | 38 |
| Nemadactylus bergi | 36 | 39 |
| Octopus tehuelchus | 37 | 40 |
| Ovalipes trimaculatus | 38 | 41 |
| Paralichthys spp | 39 | 42 |
| Parona signata | 40 | 43 |
| Percophis brasiliensis | 41 | 44 |
| Sympterygia spp | 42 | 45 |
| Triathalassothia argentina | 43 | 46 |
| Stromateus brasiliensis | 44 | 47 |
| Pinguipes brasilianus | 45 | 48 |
| Decapoda | 46 | 49 |
| Eledone massyae | 47 | 50 |
| Eurypodius latreillii | 48 | 51 |
| isopods | 49 | 52 |
| Leurocyclus tuberculosus | 50 | 53 |
| Peltarion spinosulum | 51 | 54 |
| Amphipoda | 52 | 1 |
| Cnidaria | 53 | 55 |
| Iluocoetes sp | 54 | 56 |

| Species | Fishing | Non-fishing |
|---|---|---|
| Psammobatis spp | 55 | 57 |
| Agonopsis chiloensis | 56 | 58 |
| Austropandalus grayi | 57 | 59 |
| Cottoperca gobio | 58 | 60 |
| Macruronus magellanicus | 59 | 61 |
| Majidae | 60 | 62 |
| DISCARD | 61 | NA |
| Myxine spp | 62 | 63 |
| Pagurus comptus | 63 | 64 |
| Semirossia tenera | 64 | 65 |
| Gasteropods | 65 | 66 |
| Asteroidea | 66 | 67 |
| Carcinus maenas | 67 | 68 |
| Macroalgae | 68 | 69 |
| Mysidacea | 69 | 70 |
| Robsonella fontaniana | 70 | 71 |
| Copepoda | 71 | 72 |
| Euphausiacea | 72 | 73 |
| ophiuroids | 73 | 74 |
| Peisos petrunkevitchi | 74 | 75 |
| Xystreuris rasile | 75 | 76 |
| Doryteuthis sanpaulensis | 76 | 77 |
| Diatomeas | 77 | 78 |
| Congiopodus peruvianus | 78 | 79 |
| Genypterus blacodes | 79 | 80 |
| Themisto gaudichaudii | 80 | 81 |
| Chaetognatha | 81 | 82 |
| Cyrtograpsus spp | 82 | 83 |
| Betaeus truncatus | 83 | 84 |
| Austrolycus laticinctus | 84 | 85 |
| chiton | 85 | 86 |
| Helcogrammoides cunninghami | 86 | 13 |
| Leucippa pentagona | 87 | 87 |
| Acanthistius patachonicus | 88 | 88 |
| Callorhinchus callorynchus | 89 | 89 |
| Myliobatis goodei | 90 | 90 |
| Odontesthes spp | 91 | 91 |
| Pseudopercis semifasciata | 92 | 92 |
| Gymnoscopelus nicholsi | 93 | 93 |
| Porifera | 94 | 94 |
| Salilota australis | 95 | 95 |
| Sprattus fuegensis | 96 | 96 |
| Glyceridae | 97 | 97 |
| Perumytilus purpuratus | 98 | 98 |
| Heterosquilla polydactyla | 99 | 99 |
| Nacella magellanica | 100 | 100 |
| coralline red algae | 101 | 101 |
| Pseudoechinus magellanicus | 102 | 102 |
| Cyanobacteria | 103 | 103 |
| Nematoda | 104 | 104 |
| Foraminifera | 105 | 105 |
| Lithodes santolla | 106 | 106 |
| Balanus spp | 107 | 107 |
| Dipturus chilensis | 108 | 108 |
| Mirounga leonina | 109 | 109 |
| Polyprion americanus | 110 | 110 |
| Scomber colias | 111 | 111 |

| Species | Fishing | Non-fishing |
|---|---|---|
| Seriolella porosa | 112 | 112 |
| Squalus acanthias | 113 | 113 |
| Rochinia gracilipes | 114 | 114 |
| Tegula patagonica | 115 | 115 |
| Trophon geversianus | 116 | 116 |
| Arctocephalus australis | 117 | 117 |
| Eubalaena australis | 118 | 118 |
| Lagenorhynchus obscurus | 119 | 119 |
| Notorynchus cepedianus | 120 | 120 |
| Otaria flavescens | 121 | 121 |
| Seriolella punctata | 122 | 122 |
| Mnemiopsis leidyi | 123 | 123 |
| Ascidiacea | 124 | 131 |
| Bathyraja spp | 125 | 138 |
| Cheilodactylus bergi | 126 | 133 |
| Discopyge tschudii | 127 | 139 |
| Eleginops maclovinus | 128 | 124 |
| Galeorhinus galeus | 129 | 145 |
| Schroederichthys bivius | 130 | 140 |
| Urophycis brasiliensis | 131 | 134 |
| Ramnogaster arcuata | 132 | 125 |
| Ribeiroclinus eigenmanni | 133 | 126 |
| Aulacomya atra | 134 | 127 |
| Fissurella spp | 135 | 12 |
| Bryozoa | 136 | 128 |
| Holothurians | 137 | 129 |
| Hydrozoa | 138 | 130 |
| Ostracoda | 139 | 132 |
| Odontocymbiola magellanica | 140 | 135 |
| Zearaja chilensis | 141 | 136 |
| Bassanago albescens | 142 | 137 |
| Atlantoraja cyclophora | 143 | 141 |
| Bovichthys argentinus | 144 | 142 |
| Buccinanops globulosus | 145 | 143 |
| Cephalorhynchus commersonii | 146 | 144 |
| Genypterus brasiliensis | 147 | 146 |
| Globicephala melas edwardii | 148 | 147 |
| Lagenorhynchus australis | 149 | 148 |
| Larus atlanticus | 150 | 149 |
| Larus dominicanus | 151 | 150 |
| Macronectes giganteus | 152 | 151 |
| Orcinus orca | 153 | 152 |
| FISHERY | 154 | NA |
| Phalacrocorax atriceps | 155 | 153 |
| Phalacrocorax magellanicus | 156 | 154 |
| Phalacrocorax olivaceus | 157 | 155 |
| Pleurobranchaea maculata | 158 | 156 |
| Procellaria aequinoctialis | 159 | 157 |
| Raja flavirostris | 160 | 158 |
| Renilla sp | 161 | 159 |
| Spheniscus magellanicus | 162 | 160 |
| Squatina guggenheim | 163 | 161 |
| Sterna hirundinacea | 164 | 162 |
| Tachyeres leucocephalus | 165 | 163 |
| Thalassarche melanophris | 166 | 164 |
| Thalasseus maximus | 167 | 165 |

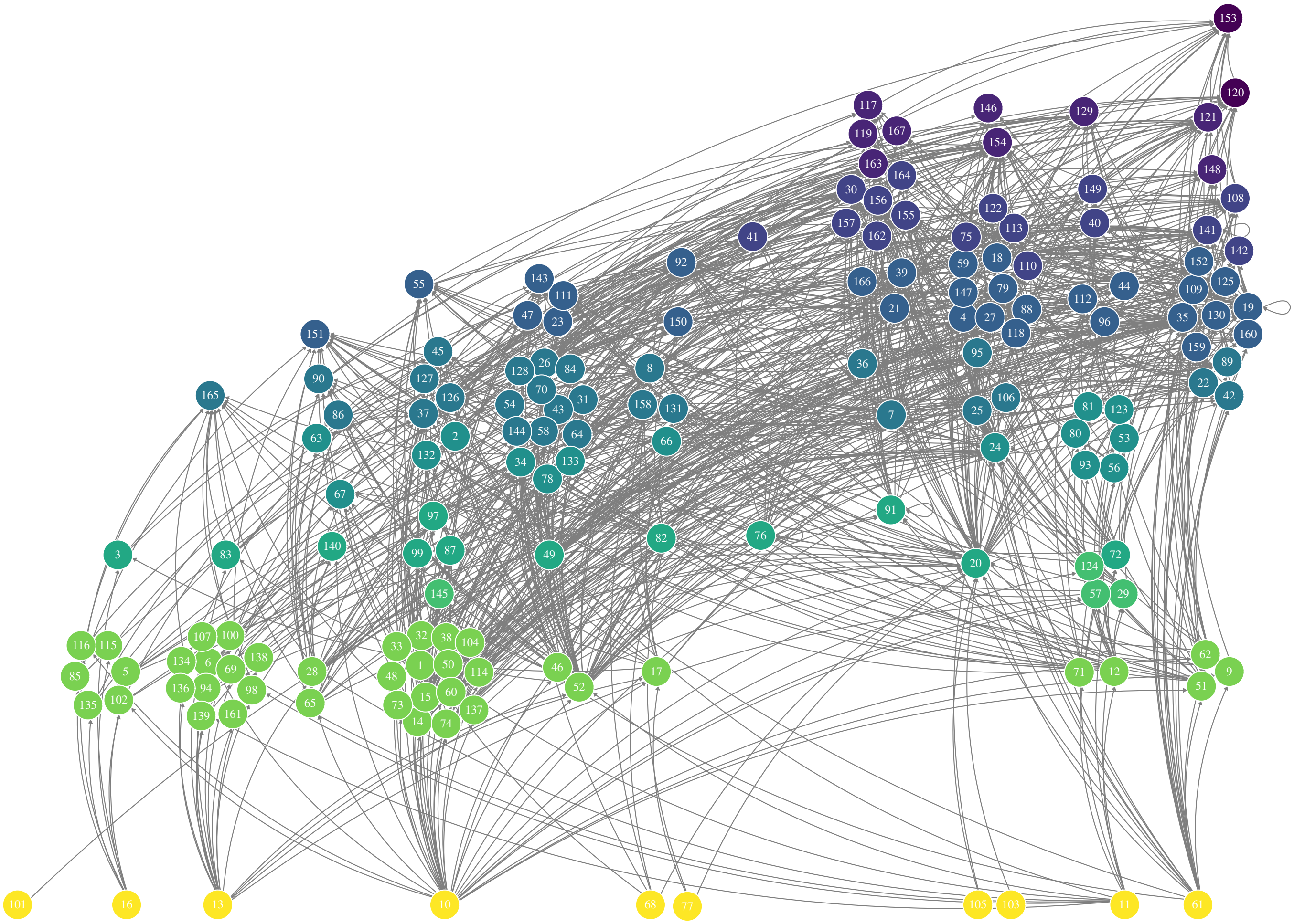


Figure S1: Fishing food web. The network is ordered and colored by trophic level in the y axis, the x axis represent different modules calculated with diet weights. Arrows represent trophic interaction between nodes, and the direction indicates energy and biomass fluxes. Check Table S2 for species references

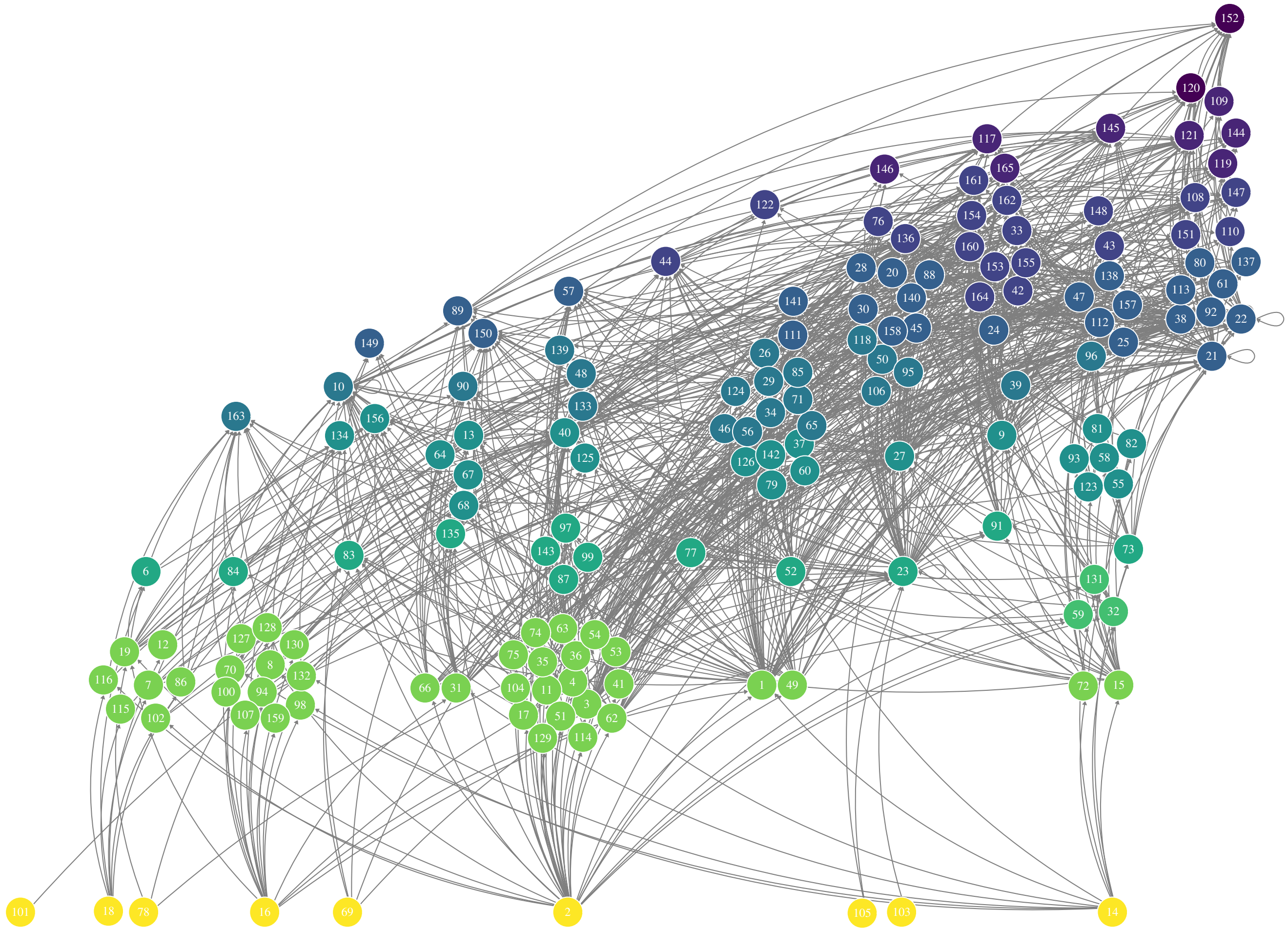


Figure S2: Non fishing food web. The network is ordered and colored by trophic level in the y axis, the x axis represent different modules calculated with diet weights. Arrows represent trophic interaction between nodes, and the direction indicates energy and biomass fluxes. Check Table S2 for species references
